# Control of Methicillin-Resistant *Staphylococcus aureus* Using Photodynamic Therapy in Synergy with *Staphylococcus epidermidis*: Role of Mixed Cultures in Developing Strategies to Inhibit Infections

**DOI:** 10.3390/microorganisms13061196

**Published:** 2025-05-23

**Authors:** Rebeca Vieira de Lima, Kate Cristina Blanco, Vanderlei Salvador Bagnato

**Affiliations:** 1 Biotechnology Postgraduate Program (PPG Biotec), Federal Univesity of São Carlos, São Carlos 13565-905, SP, Brazil; 2 São Carlos Institute of Physics (IFSC), University of São Paulo (USP), São Carlos 13566-590, SP, Brazil; kateblanco@ifsc.usp.br (K.C.B.); vander@ifsc.usp.br (V.S.B.); 3 Department of Biomedical Engineering, Texas A&M University, College Station, TX 77843, USA

**Keywords:** microbiota, photodynamic inactivation, bacterial resistance, infections, *Staphylococcus*

## Abstract

*Staphylococcus aureus* is a Gram-positive bacterium living abundantly on our skin and mucous membranes. When there is an imbalance in microbiota, they are the main protagonists of various infections, such as soft tissue infections and bacteremia. However, *Staphylococcus epidermidis* also colonizes this microbiome, is able to compete with pathogenic bacteria, including methicillin-resistant *Staphylococcus aureus* (MRSA), and can contribute to treatments such as photodynamic inactivation (PDI) by inhibiting infection progression and restoring a healthy microbiota. In vitro photodynamic inactivation experiments were carried out using synthetic curcumin at a concentration of 5 μM as a photosensitizer and varying light doses (1, 2 and 5 J/cm^2^) at a wavelength of 450 nm, on pure cultures (*S. aureus*, *S. epidermidis* and MRSA) and mixed cultures, in which bacteria were placed together proportionally. This study revealed that pure cultures of these bacteria obtained statistically significant results with varying light doses of 2 and 5 J/cm^2^. In addition, in an attempt to bring infections closer to reality, experiments were carried out on mixed cultures. The results were not only significant but also increased reduction of bacteria, including resistant bacteria. Study offers new perspectives on the importance of themicrobiota for treatment of infections caused by the *Staphylococcus* genus.

## 1. Introduction

Several therapeutic approaches have been used to treat infections, including phage therapy. However, this strategy is often limited by the rapid development of bacterial resistance, potential systemic side effects, possible intestinal dysfunction, the need for combination with other treatments, and challenges in producing well-defined and regulated phage preparations. Another alternative is the use of topical probiotics, which face drawbacks such as strain instability, the requirement for specific formulations, and individual variability among patients—particularly those with atopic dermatitis, a condition associated with skin barrier dysfunction and microbial imbalance. In such cases, there is an increase in colonization with bacteria such as *S. aureus*, which exacerbates symptoms Additional therapies include sodium hypochlorite baths, whose efficacy remains uncertain. These baths do not fully eradicate *S. aureus*, may cause cellular toxicity and irritation, and are often no more effective than plain water. These approaches, among others, still heavily rely on antibiotics.

However, the intensive use of antibiotics poses significant limitations, such as the selection of resistant bacteria and the negative impact on healthy microbiota [1]. The overuse of broad-spectrum antibiotics can disrupt eubiosis, favoring the proliferation of resistant pathogens such as methicillin-resistant *Staphylococcus aureus* (MRSA) and diminishing the population of protective bacteria [2]. This dysbiosis creates an environment conducive to the growth of pathogenic microorganisms and toxin release, leading to chronic inflammation, which contributes to disease progression and is considered a key factor in carcinogenesis. Therefore, considering the impacts on the microbiota is crucial for the development of truly effective treatments [3,4,5]. Nanotechnology-based antibacterial therapies and PDI represent effective and biocompatible alternatives for combating and treating resistant infections [6,7,8,9]. Nanotechnology leverages the unique properties of nanomaterials to enhance treatments such as photodynamic therapy. Recent studies have shown that gold quantum dots (AuQDs) exhibit potent antimicrobial activity and low cytotoxicity, being effective against methicillin-resistant *Staphylococcus aureus* and other microorganisms. When combined with femtosecond laser irradiation, AuQDs demonstrate a synergistic effect, surpassing the efficacy of each treatment individually [10]. In parallel, another study showed that femtosecond laser irradiation alone was also promising, significantly reducing *S. aureus* viability and extending its latency phase [11]. PDI has emerged as a promising treatment for modulating the microbiota and combating resistant pathogens. It overcomes the limitations of antibiotics by disrupting bacterial resistance mechanisms, preventing selection for resistant strains, and preserving host tissues [12,13,14,15,16]. PDI involves a photosensitizer (PS) that, when activated by visible light, transfers energy to molecular oxygen, producing reactive oxygen species (ROS) such as singlet oxygen, hydrogen peroxide, and hydroxyl radicals. These ROS induce irreversible oxidative damage to pathogenic cells.

*Staphylococcus* species have a cell wall composition rich in peptidoglycan and lipoteichoic acids, making them preferential absorbers of anionic photosensitizers such as curcumin—extracted from *Curcuma longa*—thus making them more susceptible to PDI [17]. This susceptibility of *Staphylococcus aureus* to PDI is mainly due to its cell wall composition, which notably lacks an outer membrane, classifying it as a Gram-positive bacterium [18,19]. This pathogen proliferates extensively and is associated with a wide range of infections, from mild skin and wound infections to precancerous lesions and even fatal sepsis [20,21,22,23]. It is also commonly found in various infectious oral conditions such as angular cheilitis, parotitis, and staphylococcal mucositis [24]. Like other bacteria, *S. aureus* can acquire resistance genes via horizontal gene transfer, contributing to the emergence of multidrug-resistant strains such as MRSA, a leading nosocomial pathogen [25]. This trend is a major global health concern due to increased treatment complexity and costs [26]. In contrast, some commensal species such as *Staphylococcus epidermidis* play beneficial roles. Though opportunistic, *S. epidermidis* can inhibit *S. aureus* colonization and produce extracellular serine protease (ESP), which degrades *S. aureus* biofilms. Identifying and utilizing beneficial *S. epidermidis* strains in combination with PDI may enhance therapeutic outcomes [27]. More studies are needed to identify potential *S. epidermidis* strains and conduct complementary experimental investigations, particularly exploring their promising application in combination with photodynamic therapy. While the use of pure cultures and the analysis of individual bacterial species are essential for characterizing microbial properties, this approach alone does not accurately reflect the complexity of natural microbial ecosystems. Studying mixed cultures, on the other hand, is essential for a more realistic understanding of microbial interactions, such as those occurring in the human microbiota [28]. The microbiota includes diverse species that interact with each other and the host, influencing processes like nutrient competition, chemical signaling, and resistance to antimicrobial agents [29]. Studies involving mixed cultures provide deeper insights into these interactions and how external factors—such as antibiotics and photodynamic therapies—can modulate microbial communities. Therefore, research incorporating mixed cultures not only expands our understanding of microbial dynamics, but also contributes, through photodynamic inactivation, to the development of more effective strategies for infection control and microbiota modulation in various contexts. Despite the growing literature on antimicrobial photodynamic therapy, there is a significant gap regarding how commensal microorganisms can influence its efficacy, particularly in polymicrobial environments. The novelty of the present study lies in the inclusion of *Staphylococcus epidermidis*, a skin commensal bacterium, in mixed cultures with pathogenic *Staphylococcus aureus* and methicillin-resistant *S. aureus* (MRSA), aiming to mimic microbial ecosystems closer to reality. Our motivation stems from the hypothesis that the presence of beneficial bacteria may not only modulate the susceptibility of pathogens to PDI, but also contribute to microbiota restoration. By combining PDI with principles of ecological microbiology, this research aims to pioneer a therapeutic strategy targeting resistant pathogens while promoting microbiome homeostasis.

## 2. Materials and Methods

### 2.1. Strains and Pre-Inoculum Preparation

*Staphylococcus aureus* (ATCC 25923), *Staphylococcus epidermidis*, and methicillin-resistant *Staphylococcus aureus* (MRSA) were thawed and streaked onto petri dishes containing Brain Heart Infusion agar (BHI agar). The plates were then incubated at 37 °C for 24 h under aerobic conditions. After the incubation period, bacterial colonies were suspended in 10 mL of Brain Heart Infusion (BHI) broth and incubated at 37 ± 2 °C with orbital shaking at 150 revolutions per minute (rpm) (Quimis Scientific Apparatus LTDA, Diadema, SP, Brazil) overnight.

### 2.2. Light Source—Biotable

**Figure 1:**
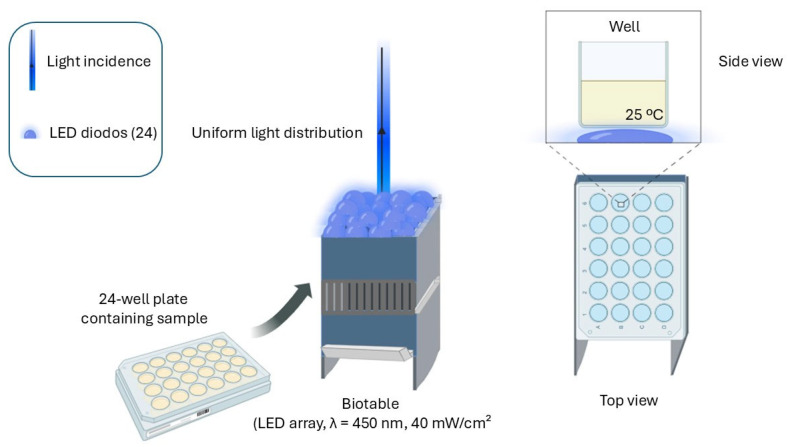
Diagram of the experimental irradiation setup used for inactivation tests. A 24-well plate containing the samples was placed in the custom-made Biotable device, equipped with a set of 24 blue LEDs (λ = 450 nm, irradiance = 40 mW/cm^2^). The LED system ensures an even distribution of light in all the wells. A side view of a single well illustrates the irradiation condition (at room temperature 25 °C). The configuration allows for consistent and homogeneous exposure to light, guaranteeing the reproducibility of the photoinactivation experiments. Image produced by BioRender.

The Biotable is a device developed by the Technological Support Laboratory of the Institute of Physics of São Carlos—University of São Paulo (USP) (Figure 1). It consists of 24 light-emitting diodes (LEDs) with a wavelength of 450 nm. The power output is 116 watts, measured using a potentiometer (Coherent Electronic Power Meter—model LabMax-TOP, Saxonburg, PA, USA) with detectors approximately 1.98 cm in diameter and an area of 2.9 cm^2^. Accordingly, the irradiance per cm^2^ was calculated using the following formula:

(1) $$I=\frac{P}{A}$$

I = Irradiance; P = Power of incident radiation, measured by the power meter; and A = Area of the power meter detector.

Accordingly, each sample was uniformly irradiated with an average intensity of 40 mW/cm^2^. The light doses (fluence) applied were 1 J/cm^2^, 2 J/cm^2^, and 5 J/cm^2^, which correspond to irradiation times of 25 s, 50 s, and 2 min, respectively, calculated using the following equation:

**D** = **i** × **T** (2)

D = light dose, expressed in J/cm^2^; I = light intensity, expressed in W/cm^2^; and T = time, given in seconds.

### 2.3. Photodynamic Inactivation (PDI)

Experiments were conducted in both pure and mixed cultures, where bacteria were combined in equal proportions. Additionally, preliminary tests were performed in triplicate using different bacterial ratios, such as 20%/80% and 80%/20%. For sample preparation, pre-inocula were centrifuged at 3000 rpm, and 1 mL of the bacterial suspension was resuspended in 9 mL of phosphate-buffered saline (PBS). The suspensions were then adjusted to 10^8^ CFU/mL at 600 nm using a spectrophotometer (Cary UV-Vis50, Varian, Polo Alto, CA, USA). After standardizing the PDI protocol, binary mixed-culture inocula were prepared with 50% of each bacterial species, while ternary mixed cultures contained 33.33% of each bacterium, and were subsequently added to 9 mL of PBS. Control groups were prepared as follows: Control (bacteria + PBS), Photosensitizer (PS) control (bacteria + photosensitizer), and Light (bacteria + illumination). For the treatment group (PDI), 250 μL of bacterial suspension and 250 μL of PBS were transferred into a well of a multiwell plate. PDI treatment involved the use of a photosensitizer (PS) to determine the optimal experimental concentration. From a 5 mM stock solution of synthetic curcumin (PDT Pharma Pharmaceutical Industry and Commerce Ltda, Cravinhos, SP, Brazil), preliminary tests were performed in triplicate using concentrations of 5 μM, 1 μM, and 0.75 μM, diluted in PBS. Irradiation was carried out using a 450 nm LED device (Biotable—developed by the Technological Support Laboratory of the Institute of Physics of São Carlos—University of São Paulo, São Carlos, SP, Brazil (USP)).

In preliminary tests, a light dose of 10 J/cm^2^ was used. However, to achieve both partial and complete bacterial reduction, the selected light doses for the experiments were 1 J/cm^2^, 2 J/cm^2^, and 5 J/cm^2^. Following treatment, samples were plated on BHI agar and incubated in a biochemical oxygen demand (BOD) incubator for 24 h, and colony-forming units (CFU/mL) were counted. These tests allowed the determination of optimal PDI conditions to achieve effective bacterial reduction for each experimental group. The uncertainty in light intensity measurements from the LED irradiation system (Biotable) was estimated to be approximately 10%, based on repeated calibration procedures using a Coherent Electronic Power Meter—model LabMax-TOP, Saxonburg, PA, USA power meter. This variability stems from inherent fluctuations in LED output and detector alignment. Despite this, uniform sample positioning and standardization of exposure time were employed to minimize inter-sample variation and ensure consistent fluence delivery across all experimental replicates.

### 2.4. Statistical Analysis

Experiments were performed in triplicate (N = 9). The results were analyzed by ANOVA (one-way) associated with post hoc Tukey test, error bars were determined by standard deviation, and a *p* value > 0.05 was considered statistically significant.

## 3. Results

### 3.1. Results of Photodynamic Inactivation Standardization for Bacteria

**Figure 2:**
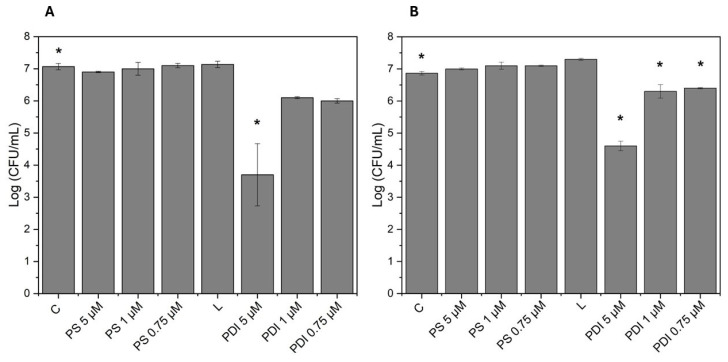
Bacterial reduction graphs from tests with different proportions of mixed cultures: (**A**)—20% *Staphylococcus aureus* + 80% *Staphylococcus aureus* resistant to methicillin (MRSA) and (**B**)—80% *Staphylococcus aureus* + 20% *Staphylococcus aureus* resistant to methicillin (MRSA), varying the concentration of the photosensitizer curcumin at 5 μM, 1 μM, and 0.75 μM, using a light dose of 10 J/cm^2^. C—Control group; PS—Photosensitizer controls (PS 5 μM), (PS 1 μM), (PS 0.75 μM); L— Light control = 10 J/cm^2^; and PDI—Photodynamic inactivation treatment groups, (PDI 5 μM), (PDI 1 μM), (PDI 0.75 μM). Calculated by mean and standard deviation, *—A *p* value > 0.05 was considered statistically significant.

Several tests were conducted to characterize and standardize photodynamic inactivation (PDI) for the bacteria under study. We began by testing *Staphylococcus aureus* and methicillin-resistant *Staphylococcus aureus* (MRSA) in different proportions of mixed cultures—20% *Staphylococcus aureus* + 80% MRSA (Figure 2A) and the inverse, 80% *Staphylococcus aureus* + 20% MRSA (Figure 2B). Additionally, these proportions were tested with different concentrations of photosensitizer (synthetic curcumin), and all experiments used a light dose of 10 J/cm^2^. Control groups were prepared as follows: Control (bacteria + PBS), PS control (bacteria + PS) for each curcumin concentration tested (5 μM, 1 μM, and 0.75 μM), Light control (bacteria + PBS), and treatment groups with PDI, varying the PS concentrations. Figure 2 shows the bacterial reduction on a logarithmic scale, measured in colony-forming units (CFU/mL) (*y*-axis) for each group (*x*-axis). The results for the mixed culture in both proportions indicate that the Control (C) group maintained high bacterial counts, the PS control (PS) group did not show significant reduction, indicating that the photosensitizer alone at these concentrations did not exhibit a direct antimicrobial effect, and the Light control (L) group also showed no significant effect, reinforcing that this light dose alone did not inactivate the bacteria. In the PDI treatment groups, however, there was a statistically significant bacterial reduction (approximately 3.5 logs) only in the PDI group using 5 μM with the mixed culture in the proportion of 20% *Staphylococcus aureus* + 80% MRSA (Figure 2A). In the inverse proportion (Figure 2B), the results were statistically significant for all PDI treatment groups across all tested PS concentrations. For the PDI 5 μM group, the reduction was about 2.4 logs, while for the other concentrations, the reduction was around 0.5 log. Thus, it was determined that PDI with synthetic curcumin is effective for photodynamic inactivation, especially at higher concentrations, such as 5 μM, which was chosen as the experimental photosensitizer concentration. Additionally, we concluded that the proportion of *S. aureus* and MRSA in the cultures influences the treatment response. However, when using the appropriate photosensitizer concentration, we were able to achieve statistically significant results, ensuring the treatment’s effectiveness. Therefore, neither light alone nor the photosensitizer alone reduces bacterial viability, highlighting that the combination is essential for inactivation.

**Figure 3:**
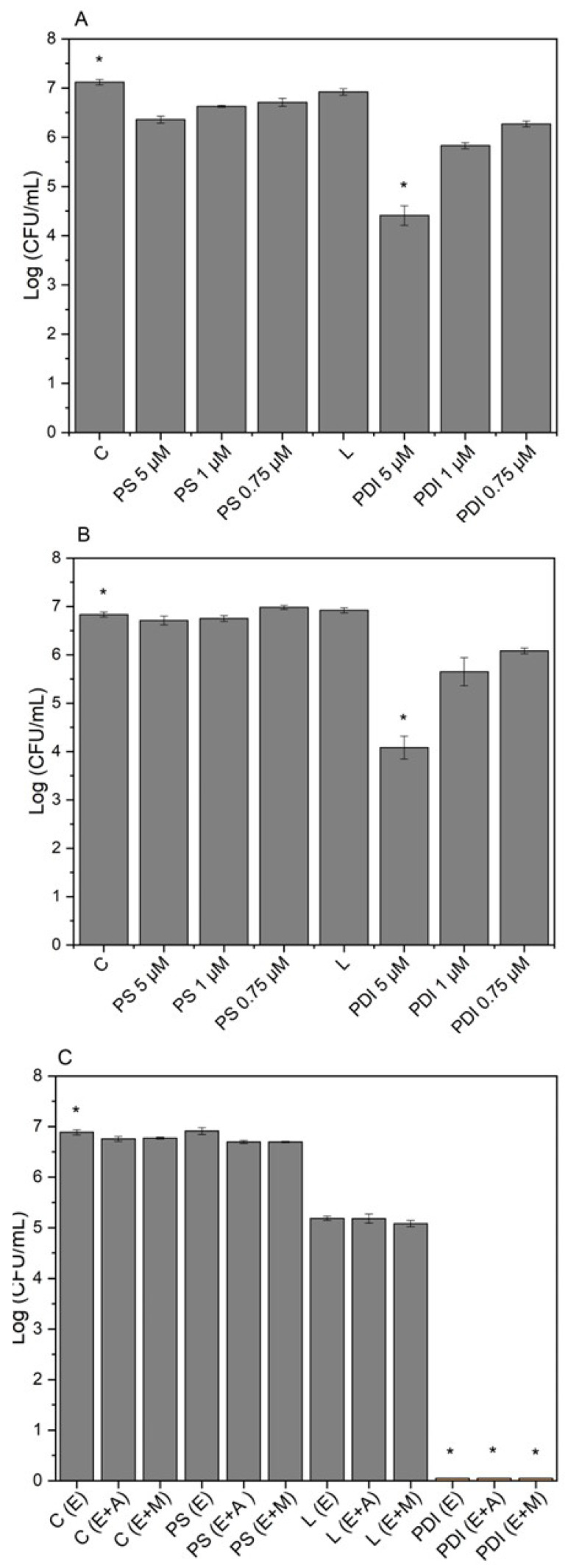
Graphs of preliminary assays testing pure cultures of (**A**)—*Staphylococcus aureus* and (**B**)—methicillin-resistant *Staphylococcus aureus* (MRSA), varying the photosensitizer concentration at 5 μM, 1 μM, and 0.75 μM. C—Control group; PS—Photosensitizer controls, (PS 5 μM), (PS 1 μM), (PS 0.75 μM); L—Light control = 10 J/cm^2^; and PDI—Photodynamic inactivation treatment groups, (PDI 5 μM), (PDI 1 μM), (PDI 0.75 μM). Inclusion of the strain (**C**)—*Staphylococcus epidermidis* and its mixed cultures (E + A) *S. epidermidis* + *S. aureus* and (E + M) *S. epidermidis* + MRSA, at equal proportions of 50% each, using only the 5 μM photosensitizer concentration. All assays used a light dose of 10 J/cm^2^. Calculated by mean and standard deviation. *—A *p* value > 0.05 was considered statistically significant.

Thus, variations in the photosensitizer concentration were also tested in pure cultures of *Staphylococcus aureus* (Figure 3A) and MRSA (Figure 3B). Additionally, the *Staphylococcus epidermidis* strain (Figure 3C) and its mixed cultures were included in the tests, now at an equivalent ratio of 50% of each bacterium, using only a 5 μM concentration of photosensitizer. For all groups, the light dose used was 10 J/cm^2^. The results were statistically significant for all tests at the 5 μM synthetic curcumin concentration. However, for *S. epidermidis*, total bacterial reduction was observed, and it was necessary to test different light doses.

**Figure 4:**
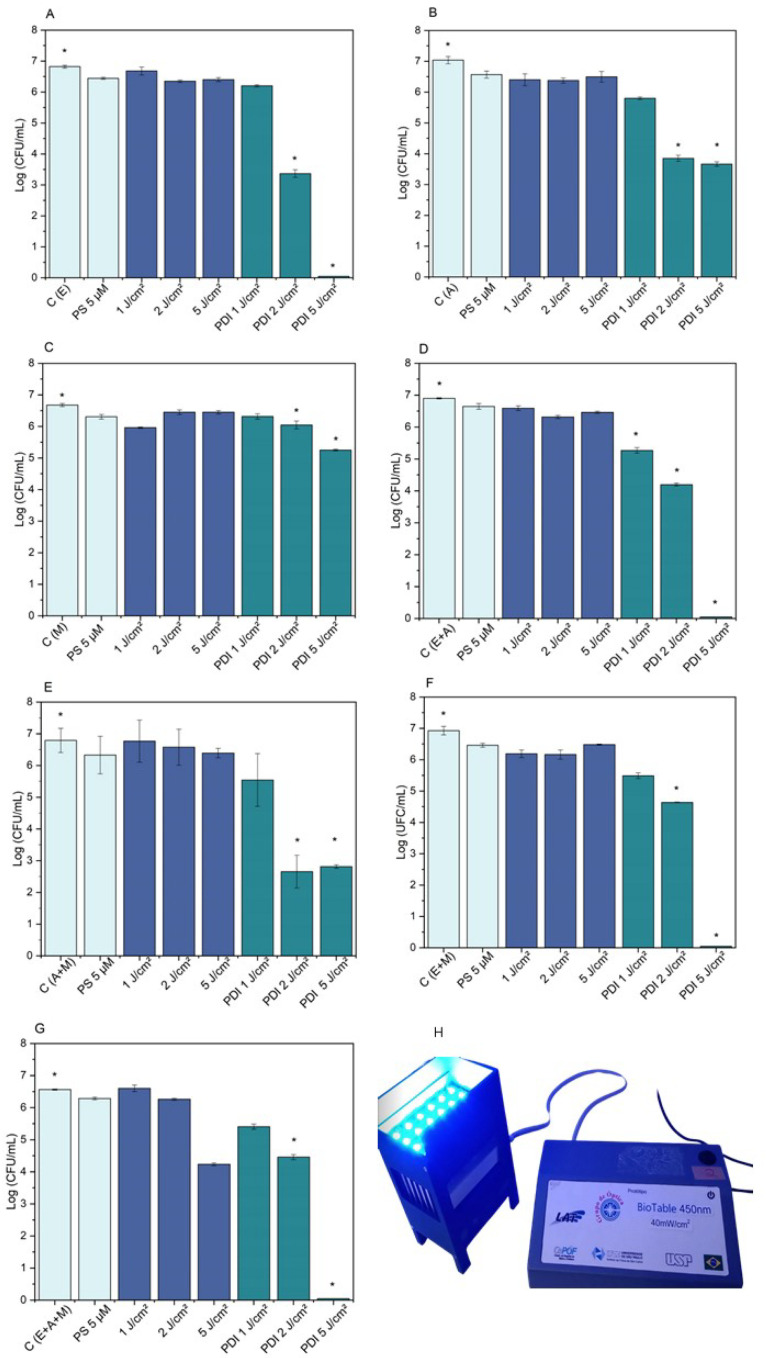
Graphs of bacterial reduction on a logarithmic scale (CFU/mL) by light dose (1, 2, and 5 J/cm^2^) for pure bacterial cultures: (**A**)—*Staphylococcus epidermidis*, (**B**)—*Staphylococcus aureus*, and (**C**)—*Staphylococcus aureus* resistant to methicillin (MRSA), and for mixed cultures at a 50% proportion of each bacterium: (**D**)—*S. epidermidis* + *S. aureus*, (**E**)—*S. aureus* + MRSA, (**F**)—*S. epidermidis* + MRSA, and mixed culture (**G**)—containing all three strains together—at a 33.33% proportion of each bacterium. (**H**)—LED lighting device (Biotable—developed by the Technological Support Laboratory of the Institute of Physics of São Carlos—University of São Paulo (USP)) emitting a wavelength of 450 nm, used to vary light doses of 1 J/cm^2^, 2 J/cm^2^, and 5 J/cm^2^. C—Control group; PS—Photosensitizer control, (PS 5 μM); L—Light controls, (1 J/cm^2^), (2 J/cm^2^), and (5 J/cm^2^); and PDI—Photodynamic inactivation treatment groups, (PDI 1 J/cm^2^), (PDI 2 J/cm^2^), and (PDI 5 J/cm^2^). Calculated by mean and standard deviation. *—A *p*-value > 0.05 was considered statistically significant.

The photosensitizer concentration was set at 5 μM, and the proportion was always kept equivalent for the mixed cultures. However, the first light dose tested was 10 J/cm^2^, which was not ideal because, despite good bacterial reduction of *S. aureus* and MRSA, it completely killed *S. epidermidis*, an undesirable effect. Therefore, based on the experience of our research group, we decided to vary it by testing light doses of 5 J/cm^2^ and lower doses of light, such as 1 J/cm^2^ and 2 J/cm^2^. In this way, we observed the responses of the bacterial strains we worked with and arrived at the ideal dose, at which photodynamic action would be guaranteed, reducing the bacteria by around 3 logs. And if it were necessary to completely contain the infection, we could irradiate with 5 J/cm^2^ or 10 J/cm^2^ (dose for inactivating mixed cultures with MRSA). Consequently, different light doses (1 J/cm^2^, 2 J/cm^2^, and 5 J/cm^2^) were tested using the Biotable—developed by the Technological Support Laboratory of the Institute of Physics of São Carlos—University of São Paulo, São Carlos, SP, Brazil (USP)). equipment (Figure 4H). As shown in Figure 4, the graphs present bacterial reduction in colony-forming units (CFU/mL) on a logarithmic scale, considering different experimental conditions: pure cultures of *Staphylococcus aureus* (Figure 4B), *Staphylococcus epidermidis* (Figure 4A), and MRSA (Figure 4C), mixed cultures in a 50% proportion of each, *S. epidermidis* + *S. aureus* (Figure 4D), *S. aureus* + MRSA (Figure 4E), *S. epidermidis* + MRSA (Figure 4F), and, in a 33.33% proportion of each, *S. epidermidis* + *S. aureus* + MRSA (Figure 4G). The results were statistically significant for all groups at light doses of 2 and 5 J/cm^2^, and only for the *S. epidermidis* + *S. aureus* group (Figure 4D) was the light dose of 1 J/cm^2^ also statistically significant.

### 3.2. Survival Curve

**Figure 5:**
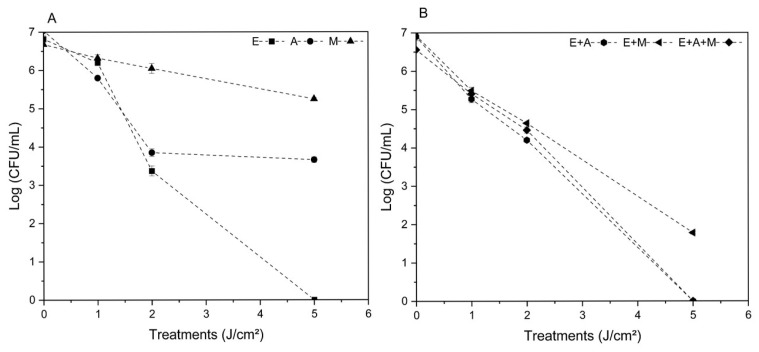
Survival curve by light dose (1, 2, and 5 J/cm^2^) of pure bacterial cultures (**A**): E—*Staphylococcus epidermidis*, A—*Staphylococcus aureus*, and M—methicillin-resistant *Staphylococcus aureus* (MRSA), and mixed cultures (**B**): *S. epidermidis* and *S. aureus* (E + A), *S. epidermidis* and MRSA (E + M), and containing all three strains together (E + A + M). Calculated by mean and standard deviation.

The results of photodynamic inactivation (PDI) conducted in the BioPhotonics Laboratory—IPSC—USP, demonstrated (Figure 5) the relationship between the applied light doses (1 J/cm^2^, 2 J/cm^2^, and 5 J/cm^2^, *x*-axis) and the colony-forming unit (CFU/mL, *y*-axis) count on a logarithmic scale, indicating cell survival. Figure 5A represents the bacterial group of pure cultures. It is observed that *Staphylococcus epidermidis* (E, squares) showed the highest reduction in bacterial count, being eliminated at 5 J/cm^2^. However, at 2 J/cm^2^, survival rate control was achieved with a reduction of approximately 3 logs. *Staphylococcus aureus* (A, circles) also showed a significant reduction but maintained a residual level of viable bacteria in pure culture. The MRSA group (M, triangles), being a methicillin-resistant strain, was less affected, maintaining a higher survival curve throughout the treatment, suggesting difficulty in treating this resistant bacterium in pure culture, even with the increase in treatment dose. On the other hand, in Figure 5B, representing mixed bacterial cultures, the data indicate that the response to PDI may be influenced by the interaction between different bacterial species. This can be observed in the combination of *S. epidermidis* and *S. aureus* (E + A), where the treatment became more effective as the light dose increased. In the combination of *S. epidermidis* and MRSA (E + M), the reduction was less pronounced, possibly due to the resistance of this strain. However, when in mixed culture, the treatment was more efficient, even in the combination of all three strains together (*S. epidermidis*, *S. aureus*, and MRSA, E + A + M), where the CFU/mL count showed a significant decrease as the light dose increased to 5 J/cm^2^. Therefore, these preliminary results reinforce the feasibility of the proposed approach and indicate that the effectiveness of PDI can be modulated by the bacterial composition present in the microenvironment. Thus, the next experiments aim to further characterize these effects, evaluating additional parameters such as photosensitizer internalization, cell viability, and potential interactions with tumor cells.

## 4. Discussion

### 4.1. About Standardization of Photodynamic Inactivation

The results obtained demonstrated the importance of standardization for the effectiveness of photodynamic inactivation (PDI) of *Staphylococcus aureus*, methicillin-resistant *Staphylococcus aureus* (MRSA), and *Staphylococcus epidermidis*. Initially, we faced difficulties in standardizing the concentrations of the photosensitizer and light doses. We chose to test how each component of the photodynamic action interacted with the conditions tested and analyzed their responses. Considering the mechanism of action of photodynamic inactivation, irradiation at a wavelength of 450 nm, for example, is important due to its ability to stimulate the production of reactive oxygen species (ROS) in photosensitizing molecules such as curcumin. The photosensitizer absorbs blue light (such as that emitted by a 450 nm LED) and transfers energy to oxygen, forming ROS, such as singlet oxygen, which destroys bacteria. The irradiation time is also a very important parameter in photodynamic inactivation, as it directly influences the total amount of light energy delivered to the system. This affects the generation of reactive oxygen species (ROS). Basically, the longer the exposure time, the greater the production of ROS, up to a certain limit, resulting in greater microbial inactivation or greater cellular damage. The limit is related to the depletion of local oxygen, the photodegradation of the photosensitizer, and the maximum effect achieved in terms of cell death. In short, the ideal irradiation time is the one that has an ideal light dose capable of generating sufficient ROS for inactivation, without causing adverse effects due to excess energy. We also encountered difficulties in standardizing PDI when testing mixed cultures. This highlights how living microorganisms have different mechanisms that generate varied responses, emphasizing the need for caution in developing truly effective treatments [30]. For example, mixed cultures revealed differences in treatment response depending on *S. aureus* and MRSA proportions. When culture contained 20% *S. aureus* and 80% MRSA, only the higher PS concentration (5 μM) resulted in a significant reduction in bacterial load (about 3.5 logs). However, in the inverse situation (80% *S. aureus* and 20% MRSA), all PS concentrations tested promoted statistically significant reductions, especially the 5 μM PS concentration, resulting in a reduction of approximately 2.4 logs—a closer result to the inverse bacterial concentration group. This may be due to variations in resistance factor expression and protective mechanisms against oxidative stress [31,32,33]. Nonetheless, the overall effectiveness of PDI with synthetic curcumin [34,35,36], especially at 5 μM, shows that this strategy is promising for controlling infections caused by MRSA, regardless of the *S. aureus* proportion present. Therefore, the data presented consolidate PDI as a viable approach for the inactivation of MRSA, emphasizing the importance of selecting an appropriate PS concentration to maximize treatment efficacy. Future studies may explore PDI in combination with other antimicrobial strategies to further enhance therapy effects and reduce the viability of resistant bacteria in clinical and hospital settings. It is worth noting that the lack of bacterial reduction in the Control group (bacteria + PBS), PS control group (bacteria + PS), and Control light group (bacteria + PBS) confirms that neither light alone nor photosensitizer without activation is able to significantly inactivate bacteria, reinforcing the idea that a combination of light and PS is necessary for PDI to be effective. This corroborates previous studies that indicate dependence on reactive oxygen species (ROS) generation mechanisms for antimicrobial action [37,38]. The statistical reduction confirms importance of the correct light dose combination and photosensitizer concentration for this bacterial strain [39,40,41].

### 4.2. About Survival Curve

The results from the survival curve (Figure 5A) indicated the *S. epidermidis* exhibited the greatest reduction in bacterial count, being eliminated at 5 J/cm^2^. However, at 2 J/cm^2^, it was possible to control survival rate, with a reduction of approximately 3 logs. The *Staphylococcus aureus* strain also showed a significant reduction, but a residual level of viable bacteria remained in pure culture. In contrast, the methicillin-resistant *Staphylococcus aureus* (MRSA) group was less affected in pure culture, maintaining a higher survival curve throughout treatment. This suggests greater difficulty in treating this resistant bacterium, even with an increased light dose. On the other hand, in Figure 5B, representing mixed bacterial cultures, the data suggest that the response to PDI may be influenced by interactions between different bacterial species [42]. This is evident in the *S. epidermidis* and *S. aureus* (E + A) combination, where treatment became more effective as the light dose increased. In the *S. epidermidis* and MRSA (E + M) combination, the reduction was less pronounced, possibly due to the resistance of this strain. However, treatment demonstrated increased effectiveness when bacteria were present in a mixed culture, especially when all three strains were combined (*S. epidermidis*, *S. aureus*, and MRSA—E + A + M). In this condition, the CFU/mL count showed a substantial decrease as the light dose was increased to 5 J/cm^2^. Therefore, these results approach proposed viability and indicate that PDI effectiveness can be modulated by the bacterial composition present in the microenvironment. Consequently, the next experiments aim to further characterize these effects by evaluating additional parameters such as photosensitizer internalization, cell viability, and potential interactions with tumor cells.

## Data Availability

The original contributions presented in this study are included in the article. Further inquiries can be directed to the corresponding author.
